# Integrated analysis of proteome-wide and transcriptome-wide association studies identified novel genes and chemicals for vertigo

**DOI:** 10.1093/braincomms/fcac313

**Published:** 2022-11-28

**Authors:** Bolun Cheng, Peilin Meng, Xuena Yang, Shiqiang Cheng, Li Liu, Yumeng Jia, Yan Wen, Feng Zhang

**Affiliations:** Key Laboratory of Trace Elements and Endemic Diseases, Collaborative Innovation Center of Endemic Disease and Health Promotion for Silk Road Region, School of Public Health, Health Science Center, Xi’an Jiaotong University, No. 76 Yan Ta West Road, Xi’an 710061, China; Key Laboratory for Disease Prevention and Control and Health Promotion of Shaanxi Province, Health Science Center, Xi'an Jiaotong University, Xi’an 710061, China; Key Laboratory of Environment and Genes Related to Diseases of Ministry of Education of China, Health Science Center, Xi'an Jiaotong University, Xi'an 710061, China; Key Laboratory of Trace Elements and Endemic Diseases, Collaborative Innovation Center of Endemic Disease and Health Promotion for Silk Road Region, School of Public Health, Health Science Center, Xi’an Jiaotong University, No. 76 Yan Ta West Road, Xi’an 710061, China; Key Laboratory for Disease Prevention and Control and Health Promotion of Shaanxi Province, Health Science Center, Xi'an Jiaotong University, Xi’an 710061, China; Key Laboratory of Environment and Genes Related to Diseases of Ministry of Education of China, Health Science Center, Xi'an Jiaotong University, Xi'an 710061, China; Key Laboratory of Trace Elements and Endemic Diseases, Collaborative Innovation Center of Endemic Disease and Health Promotion for Silk Road Region, School of Public Health, Health Science Center, Xi’an Jiaotong University, No. 76 Yan Ta West Road, Xi’an 710061, China; Key Laboratory for Disease Prevention and Control and Health Promotion of Shaanxi Province, Health Science Center, Xi'an Jiaotong University, Xi’an 710061, China; Key Laboratory of Environment and Genes Related to Diseases of Ministry of Education of China, Health Science Center, Xi'an Jiaotong University, Xi'an 710061, China; Key Laboratory of Trace Elements and Endemic Diseases, Collaborative Innovation Center of Endemic Disease and Health Promotion for Silk Road Region, School of Public Health, Health Science Center, Xi’an Jiaotong University, No. 76 Yan Ta West Road, Xi’an 710061, China; Key Laboratory for Disease Prevention and Control and Health Promotion of Shaanxi Province, Health Science Center, Xi'an Jiaotong University, Xi’an 710061, China; Key Laboratory of Environment and Genes Related to Diseases of Ministry of Education of China, Health Science Center, Xi'an Jiaotong University, Xi'an 710061, China; Key Laboratory of Trace Elements and Endemic Diseases, Collaborative Innovation Center of Endemic Disease and Health Promotion for Silk Road Region, School of Public Health, Health Science Center, Xi’an Jiaotong University, No. 76 Yan Ta West Road, Xi’an 710061, China; Key Laboratory for Disease Prevention and Control and Health Promotion of Shaanxi Province, Health Science Center, Xi'an Jiaotong University, Xi’an 710061, China; Key Laboratory of Environment and Genes Related to Diseases of Ministry of Education of China, Health Science Center, Xi'an Jiaotong University, Xi'an 710061, China; Key Laboratory of Trace Elements and Endemic Diseases, Collaborative Innovation Center of Endemic Disease and Health Promotion for Silk Road Region, School of Public Health, Health Science Center, Xi’an Jiaotong University, No. 76 Yan Ta West Road, Xi’an 710061, China; Key Laboratory for Disease Prevention and Control and Health Promotion of Shaanxi Province, Health Science Center, Xi'an Jiaotong University, Xi’an 710061, China; Key Laboratory of Environment and Genes Related to Diseases of Ministry of Education of China, Health Science Center, Xi'an Jiaotong University, Xi'an 710061, China; Key Laboratory of Trace Elements and Endemic Diseases, Collaborative Innovation Center of Endemic Disease and Health Promotion for Silk Road Region, School of Public Health, Health Science Center, Xi’an Jiaotong University, No. 76 Yan Ta West Road, Xi’an 710061, China; Key Laboratory for Disease Prevention and Control and Health Promotion of Shaanxi Province, Health Science Center, Xi'an Jiaotong University, Xi’an 710061, China; Key Laboratory of Environment and Genes Related to Diseases of Ministry of Education of China, Health Science Center, Xi'an Jiaotong University, Xi'an 710061, China; Key Laboratory of Trace Elements and Endemic Diseases, Collaborative Innovation Center of Endemic Disease and Health Promotion for Silk Road Region, School of Public Health, Health Science Center, Xi’an Jiaotong University, No. 76 Yan Ta West Road, Xi’an 710061, China; Key Laboratory for Disease Prevention and Control and Health Promotion of Shaanxi Province, Health Science Center, Xi'an Jiaotong University, Xi’an 710061, China; Key Laboratory of Environment and Genes Related to Diseases of Ministry of Education of China, Health Science Center, Xi'an Jiaotong University, Xi'an 710061, China

**Keywords:** vertigo, proteome-wide association study, transcriptome-wide association study, chemical, gene set enrichment analysis

## Abstract

Vertigo is a leading symptom of various peripheral and central vestibular disorders. Although genome-wide association studies (GWASs) have identified multiple risk variants for vertigo, how these risk variants contribute to the risk of vertigo remains unknown. Discovery proteome-wide association study (PWAS) was first performed by integrating the protein quantitative trait loci from the dorsolateral prefrontal cortex (DLPFC) in the Banner Sun Health Research Institute dataset (*n* = 152) and GWAS summary of vertigo (*n* = 942 613), followed by replication PWAS using the protein quantitative trait loci from the DLPFC in Religious Orders Study or the Rush Memory and Aging Project dataset (*n* = 376). Transcriptome-wide association studies (TWASs) were then performed by integrating the same GWAS datasets of vertigo (*n* = 942 613) with mRNA expression reference from human fetal brain, and DLPFC. Chemical-related gene set enrichment analysis (GSEA) and Gene ontology/Kyoto Encyclopedia of Genes and Genomes pathway enrichment analyses were finally conducted to further reveal the pathogenesis of vertigo. Permutation-based empirical *P* values were calculated in PWAS, TWAS, and GSEA. By integrating the GWAS of vertigo and two independent brain proteomes from human DLPFC, three genes were identified to genetically regulate protein abundance levels in vertigo, and were not previously implicated by GWAS, including *MTERFD2* (*P*_Banner_ = 0.045, *P*_ROSMAP_ = 0.031)*, MGST1* (*P*_Banner_ = 0.014, *P*_ROSMAP_ = 0.018), and *RAB3B* (*P*_Banner_ = 0.045, *P*_ROSMAP_ = 0.035). Compared with TWAS results, we identified overlapping genes *RAB3B* (*P*_TWAS_ = 0.017) and *MTERFD2* (*P*_TWAS_ = 0.003) that showed significant associations with vertigo at both proteome-wide and transcriptome-wide levels. Chemical-related GSEA identified multiple chemicals that might be associated with vertigo, such as nickel (*P* = 0.007), glycidamide (*P* = 0.005), and proanthocyanidins (*P* = 0.015). Our study provides novel clues for understanding the biological mechanism of vertigo, and highlights several possible risks and therapeutic chemicals for vertigo.

## Teaser

Cheng *et al*. performed proteome-wide association studies to identify genes whose cis-regulated protein abundance changes in the human brain were associated with vertigo, followed by chemical-related gene set enrichment analysis. They provide novel genes (MTERFD2, MGST1, and RAB3B) into the pathogenesis of vertigo, and highlight promising chemicals for further therapeutics research.

## Graphical Abstract

**Graphical abstract:**
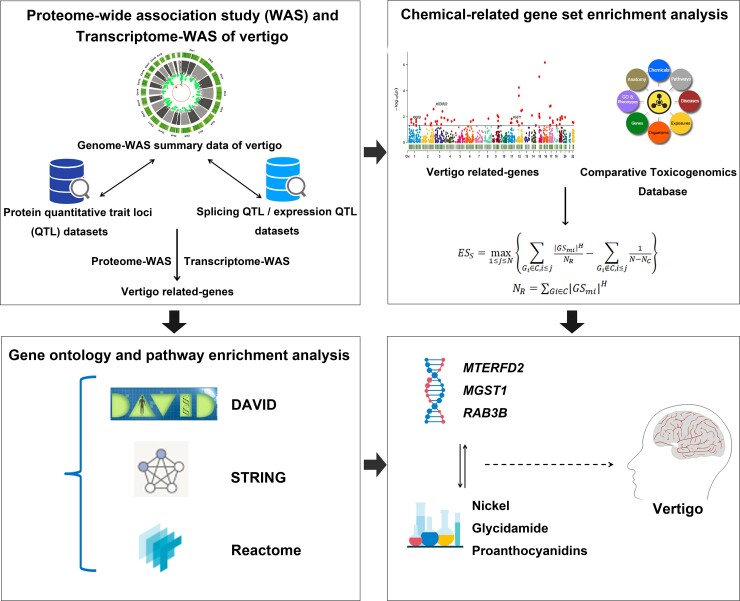

## Introduction

Vertigo is a subtype of dizziness defined as the illusion of motion caused by the asymmetrical intervention of the vestibular system.^1^ Vestibular central lesions affecting the pons, medulla, or cerebellum may cause vertigo, severe ataxia, vomiting, nausea, and other neurological signs.^1^ Conventionally, the diseases causing vertigo can be classified into three broad categories: otological vertigo, central vertigo, and psychogenic dizziness.^2,3^ Common causes of vertigo include benign paroxysmal postural vertigo, migraine, vestibular neuritis, Ménière's syndrome, adverse drug effects, and disturbed blood pressure regulation.^4^ The prevalence of vertigo is 6.5% and increases with age, with approximately 65% of patients being female^5^ and a lifetime prevalence of about 20–30%.^6^ Vertigo can come on suddenly and last for a few seconds or maybe constant for several days, which is a major risk factor for falls and bone fractures, placing a huge burden on the healthcare system.^7^

Several common vertigo syndromes are known to be genetically heterogeneous and have been identified in patients with isolated recurrent attacks of vertigo, genetic deafness syndromes, and neurological disorders.^8^ Genetic studies have identified several mutations in the *KCNA1* and *CACNA1A* genes associated with recurrent vertigo, suggesting that voltage-gated channels and solute carriers in the plasma membrane of neurons play a key role in recurrent vertigo.^9^ A genome-wide association study (GWAS) also uncovered six sequence variations associated with vertigo risk.^10^ In addition, the central nervous system (CNS) plays an important role in the pathogenesis of vertigo.^11^ Interference with protein–protein interaction networks in neurons, glia, and other cell types has also been found to be associated with multifactorial neurological disorders.^12^ Although previous research works have focused on transcription as a central regulator of protein expression, it is now increasingly recognized that the CNS relies on efficient updating of the protein landscape.^13^ However, the characteristics of brain proteins and genetics of vertigo remain unclear.

GWAS seeks to strongly link genetic loci to diseases and other heritable traits, but the statistical power is limited.^14^ Linkage disequilibrium (LD) and population stratification make it convoluted for GWAS to identify exact causal variants.^15^ The transcriptome-wide association study (TWAS) tests whether the phenotype being studied correlates with the level of gene expression predicted from genetic variants, usually requiring a target tissue for association tests.^16^ The TWAS model simulates gene expression, while the proteome-wide association study (PWAS) model simulates protein abundance, and the principle is completely orthogonal to gene abundance signals.^16^ As a new method to detect gene-phenotype associations mediated by changes in protein abundance, PWAS aggregates all variants’ signals that jointly affect protein-coding genes, reducing the burden of multiple tests and providing more interpretable discoveries.^17^ For example, Liu *et al.* performed PWAS to identify genes associated with changes in cis-regulatory protein abundance in the human brain and found several high-confidence risk proteins (including CNNM2 and CTNND1) for schizophrenia and depression.^18^ Another study performed PWAS by integrating depression GWAS and human brain proteomics, followed by Mendelian randomization, and identified 19 genes consistent with depression causality.^19^

Human brain development is disrupted by chemical exposure, leading to irreversible damage to the nervous system.^20^ Conversely, some chemicals have positive effects on the CNS.^21^ Recent studies have found that chemical exposure also affects epigenetic inheritance.^22^ For example, prenatal exposure to endocrine-disrupting chemicals might lead to poor behaviour and cognitive dysfunction in children.^23^ The contribution of chemicals to brain development is multifaceted and complex, and chemicals associated with the endocrine activity are suspected to interfere with neurodevelopment.^20^ In addition, neural tube and axial defects were frequently found when evaluating the developmental effects of chemicals in laboratory animal studies.^24^ Therefore, it is necessary to identify the chemicals related to the pathogenesis of vertigo.

In this study, PWASs were performed on vertigo by integrating a large-scale GWAS and two independent human brain protein quantitative trait loci (pQTL) datasets. Discovery PWAS was first conducted using the pQTL from the dorsolateral prefrontal cortex (DLPFC) in the Banner dataset. Replication PWAS was then performed using the pQTL from DLPFC in the ROSMAP dataset. To validate the results of PWASs, we also performed TWASs by integrating the same GWAS datasets and mRNA expression reference from the human fetal brain, and DLPFC in which gene expression was quantified with RNA-seq and RNA-seq splicing, respectively. Chemical-related gene set enrichment analysis (GSEA) and Gene ontology (GO)/Kyoto Encyclopedia of Genes and Genomes (KEGG) pathway enrichment analyses were finally conducted to further reveal the pathogenesis of vertigo. Our study identified risk genes whose protein abundance is associated with vertigo, providing novel insights into further mechanistic studies and the development of new therapies.

## Materials and methods

### GWAS summary data of vertigo

Genome-wide association summary level data used in this study was derived from a recent publicly available large-scale GWAS of human vertigo.^10^ Briefly, this dataset had 48 072 vertigo cases and 894 541 controls in the vertigo meta-analysis, all of whom were verified as being of white origin.^10^ The deCODE was used to perform the sample preparation and the whole-genome sequencing (WGS) in Iceland samples.^25^ The Affymetrix chip UK BiLEVE Axiom and the Affymetrix UK Biobank Axiom array were used to genotype the UK Biobank samples.^26^ NovaSeq Illumina was used for WGS in US samples, and Illumina Global Screening Array chips were used for genotyping. FinnGen ThermoFisher Axiom array was used to genotype FinnGen samples. The logistic regression assuming an additive model was applied and tested for association between sequence variants and vertigo using deCODE software.^25^ The fixed-effects inverse variance method based on effect estimates and standard errors was used to combine vertigo GWAS summary results from Iceland, the UK, Finland, and the US. This method assumes that all study groups have a common OR, but allows for different population frequencies for alleles and genotypes.^27^ In total, 62 056 310 variants in the meta-analysis had imputation information above 0.8 and MAF > 0.01%. The genotyping, quality control, imputation, and association analysis of the dataset have been described in detail elsewhere.^10^

### Human brain pQTL datasets for protein reference weights

Human brain proteomes datasets were obtained from a recent publicly available study.^28,29^ Briefly, a proteome analysis was performed using brain DLPFC of 376 European descent subjects collected by the Religious Orders Study (ROS) or the Rush Memory and Aging Project (ROSMAP dataset). After quality control (QC), 8356 proteins were included in proteomic profiles for pQTL analysis. By integrating proteomic data and SNP genotypes, 1475 proteins were significantly heritable to genetic variation, and their protein weights were used in our PWAS. Besides, another proteome analysis was performed using brain DLPFC of 152 European descent subjects from the Banner Sun Health Research Institute (Banner dataset).^28^ After QC, 8168 proteins were included in proteomic profiles for pQTL analysis. By integrating proteomic data and SNP genotypes, 1139 proteins were significantly heritable to genetic variation, and their protein weights were used in our PWAS. The brain protein weights from the ROSMAP and Banner brain proteome can be downloaded from https://doi.org/10.7303/syn23627957.^28^ Detailed information for the brain protein weights has been described in the study of Wingo *et al.*^28^

### PWAS for vertigo

The PWAS analysis of vertigo was performed by using FUSION software (http://gusevlab.org/projects/fusion/).^16^ Using the pre-computed reference weights of the ROSMAP and Banner brain proteome together with GWAS summary data of vertigo, FUSION is capable to estimate the associations of each protein abundance gene with vertigo. Firstly, the reference expression weights were calculated using the prediction models in FUSION. The calculated expression weights were then combined with GWAS results to impute association statistics between protein abundance gene expression levels and vertigo. Bayesian sparse linear mixed model (BSLMM) was used to compute the SNP-expression weights in the 1-Mb cis loci of the gene for a given gene.^30^ The association test statistics between predicted gene expression and vertigo were calculated as Z_PWAS_ = *W′Z*/(*W′SW*)^1/2^.^16^*Z* denotes the scores of vertigo, while *W* denotes the weights. *S* denotes the SNP-correlation covariance matrix. In this study, a total of 5000 permutation tests in FUSION were implemented to control the potential impact of multiple test problems. The permutation-based empirical *P* value was calculated for each gene within the ROSMAP and Banner brain proteome. Significant protein abundance genes were considered at permutated *P* < 0.05.

### TWAS for vertigo

The TWAS analyses were performed by using FUSION software (http://gusevlab.org/projects/fusion/).^16^ Using the pre-computed gene expression weights of different tissues together with GWAS summary data of vertigo, FUSION is capable to estimate the associations of each gene with vertigo in different tissues. In this study, TWAS analysis was performed based on the GWAS summary statistics of vertigo and pre-computed expression reference panel in TWAS included the splicing-level (splicing QTL, sQTL) and mRNA-level (eQTL) weights from CommonMind Consortium (CMC) in DLPFC profiles,^31^ and fetal brain expression (eQTL) weights.^32^ The only difference between PWASs and TWASs is the different external presentation reference panels. PWAS is based on protein abundance, while TWAS is based on mRNA expression level. Briefly, the gene expression weights were calculated using the prediction models of FUSION, respectively. The calculated expression weights were then combined with GWAS summary statistics to impute association statistics between gene expression levels and vertigo. BSLMM was used to compute the SNP-expression weights in the 1-Mb cis loci of the gene for a given gene.^30^ Multiple testing problem of each gene was computed using 5000 permutation tests. Genes with significant correlation signals were identified at permutated *P* < 0.05.

### Colocalization analysis

Colocalization analysis was performed using Fusion software with parameter—coloc *P* 0.05, which indicated that only genes with *P* < 0.05 were included to perform colocalization analysis.^16^ Colocalization of GWAS and pQTL (ROSMAP and Banner datasets),^28^ eQTL (CMC and fetal brain dataset),^32^ and sQTL (CMC dataset)^31^ signals were performed to explore the same risk variants in vertigo.

### Statistical analysis

The chemical-related gene set was obtained from the public Comparative Toxicogenomics Database (CTD) which consisted of 1 379 105 chemical–gene interactions (http://ctdbase.org/).^33^ CTD is an innovative digital ecosystem that relates toxicological information for chemicals, genes, phenotypes, diseases, and exposures to advance the understanding of human health.^34^ The GSEA (4.2.3) was implemented to evaluate the functional relevance between 10 103 chemicals and vertigo.^35^ Briefly, GSEA was first applied to the PWAS-level data of vertigo to determine whether vertigo-associated protein abundance genes were significantly enriched in the chemical-related gene sets.^35^ Following the standard GSEA approach, we set *GS_m_* as the PWAS statistic. All genes were then ordered by ranking *GS_m_* from the largest to the smallest, set as *GS_m_* = (*GS_m1_*, *GS_m2_*, …*GS_mN_*). We set *G_i_* as the *i*th gene in chemical-related gene set *C* with *N_C_* genes. A weighted Kolmogorov-Smirnov-like running sum statistic was used to calculate the enrichment scores (*ES_S_*) of each analyzed chemical.^35^

$$ES_{S}=\underset{1\leq j\leq N}{\max}\;\{\underset{G_{i}\in C,i\leq j}{\sum }\frac{{\left|GS_{mi}\right|}^{H}}{N_{R}}-\underset{G_{i}\notin C,i\leq j}{\sum }\frac{1}{N-N_{C}}\}$$

where *N_R_* defined as $\;N_{R}=\underset{Gi\in C}{\sum }\;|GS_{mi}{|}^{H}$; *N* as the total number of genes; *H* as the parameter that gives higher weights to genes with extreme statistics. We followed the recommendation that using *H* = 1 for the original GSEA algorithm. The TWAS-level data were then utilized to perform the same GSEA algorithm.

Following the normalization procedure, statistical test was performed to calculate the null distribution of *ES_S_*, with SNP labels of vertigo GWAS randomly shuffled and then used for ES calculation.^35^ The null distribution of *ES^n^* (*ES^n1^*, *ES^n2^*, *ES^n3^*, …, *ES^nt^*) was obtained after *t* time permutations. To adjust the bias caused by different sizes of chemical-related gene sets, the observed *ES_S_* was normalized by the standard deviation (Sd) and mean of permutated *ES^n^*:

$$NES_{S}=\frac{ES_{S}-mean(ES_{S}^{n})}{SD(ES_{S}^{n})}$$

Each enrichment analysis implemented 20 000 permutations. The permutation-based empirical *P* values were finally calculated using the NES for each chemical-related gene set. Significant enrichment was identified at *P* < 0.05. Detailed information of permutation and statistical analysis were described elsewhere.^36^

### Gene ontology and pathway enrichment analysis

To determine biological features and enriched pathways for vertigo, the gene ontology (GO) and pathway enrichment analysis were performed on DAVID (Dec.2021),^37^ STRING (11.5),^38^ and Reactome (Version 81)^39^ using the significant genes identified by PWAS and TWAS (*P*_PWAS_ < 0.05 or *P*_TWAS_ < 0.05), including pQTL PWAS using Banner datasets,^28^ eQTL TWAS using CMC and fetal brain dataset,^32^ and sQTL TWAS using CMC dataset.^31^ All enrichment analyses were false discovery rate (FDR) controlled using the Benjamini-Hochberg method.^40^

### Data availability

Detailed description of the datasets used in our study are shown in Supplementary Material. The datasets generated and/or analyzed in the current study are available from the corresponding authors upon reasonable request.

## Results

### PWASs identified three genes that regulate protein levels in the brain and were linked to risk of vertigo

**Figure 1:**
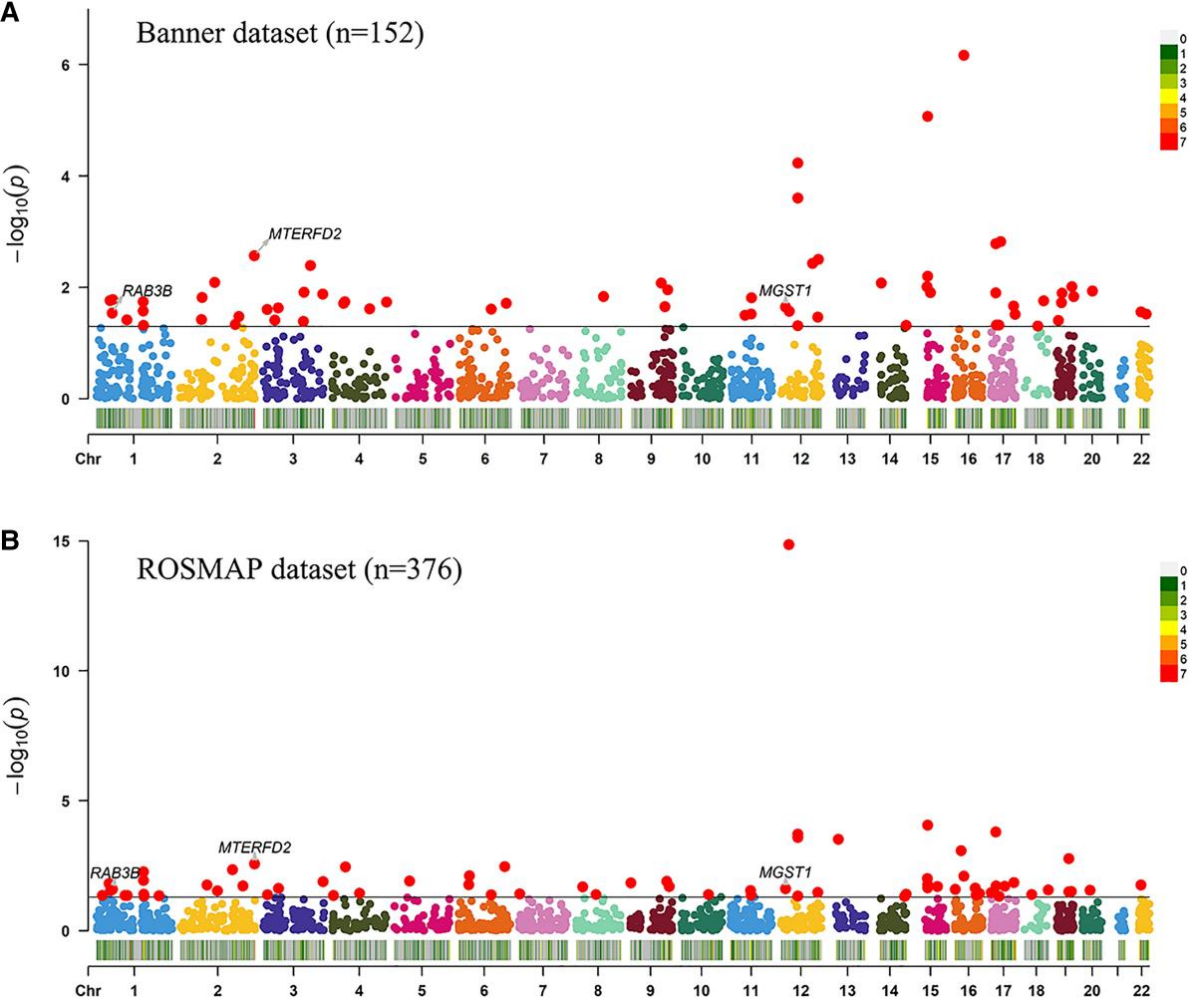
**Manhattan plots for the vertigo PWASs in the Banner and ROSMAP human brain proteomes.** (**A**) Manhattan plot for the vertigo PWAS integrating the vertigo GWAS with the Banner proteomes (*n* = 152). (**B**) Manhattan plot for the vertigo PWAS integrating the vertigo GWAS with the ROSMAP proteomes (*n* = 376). Each dot on the *x*-axis represents a gene, and the association strength on the *y*-axis represents the –log_10_(original *P* value) of PWAS. The heat-map scale bar indicates the distribution density of analyzed protein regulated genes on the chromosome. Genes that were proteome-wide significant using permutation test (*MTERFD2, MGST1,* and *RAB3B*) in Banner and ROSMAP brain proteomes are shown with mark. Chr = chromosome; PWAS = Proteome-Wide Association Study; ROSMAP = Religious Orders Study and Rush Memory and Aging Project.

Discovery PWAS was first performed by integrating the Banner human brain pQTL data and GWAS of vertigo and identified 22 significant genes for vertigo (Table 1). To validate these results, replication PWAS was then conducted using the ROSMAP human brain pQTL dataset and the same GWAS of vertigo and identified 13 significant genes for vertigo (Table 1). Importantly, three common vertigo risk genes showed proteome-wide significant signals in both discovery and replication stages, including *MTERFD2* (*P*_Banner_ = 0.045, *P*_ROSMAP_ = 0.031)*, MGST1* (*P*_Banner_ = 0.014, *P*_ROSMAP_ = 0.018), and *RAB3B* (*P*_Banner_ = 0.045, *P*_ROSMAP_ = 0.035) (Table 1). The original PWAS results based on the Banner and ROSMAP human brain proteomes are presented in Fig. 1.

**Table 1:** Proteome-wide significant genes for vertigo

Gene	CHR	Banner		ROSMAP	
		Z_PWAS_	*P*_PERM_	Z_PWAS_	*P*_PERM_
*ASPHD1*	16	4.967	2.00 × 10^−4^		
*PPP2R4*	9			−2.321	4.00 × 10^−4^
*MCEE*	2	−2.427	0.005		
*PTGR1*	9	−2.285	0.007		
*TMEM132E*	17	−3.173	0.007		
*SHISA7*	19	−2.441	0.010		
*SLC27A1*	19	2.491	0.010		
*RIMS3*	1			2.430	0.011
*MSTO1*	1	−1.97	0.011		
*ACOT11*	1	2.393	0.0119		
*NOSTRIN*	2			2.838	0.013
*CCDC91*	12			7.986	0.013
*CDH7*	18	2.376	0.014		
*MGST1* ^a^	12	2.279	0.014	2.256	0.018
*S100A1*	1			2.779	0.019
*C12orf73*	12	−2.90	0.019		
*DHRS4*	14	2.638	0.020		
*SPRYD4*	12	1.971	0.023		
*AGA*	4	−2.357	0.025		
*C2orf76*	2			2.181	0.026
*MTERFD2* ^a^	2	−2.999	0.045	−2.999	0.031
*PAK4*	19			2.147	0.032
*GPX1*	3	−2.267	0.035		
*RAB3B* ^a^	1	2.184	0.045	2.233	0.035
*COQ10A*	12	4.018	0.039		
*EXOG*	3	2.067	0.0393		
*IPCEF1*	6	−2.335	0.0396		
*PPFIA3*	19	−2.583	0.0396		
*IFIT2*	10			−2.044	0.042
*ADSL*	22	2.165	0.044		

CHR = chromosome; PERM = permutation; PWAS = proteome-wide association study; ROSMAP = Religious Orders Study and Rush Memory and Aging Project.

a Genes that reached proteome-wide significant level in both the Banner and ROSMAP brain proteomes.

### TWASs identified eight genes that regulate gene levels in the brain and were linked to risk of vertigo

The eQTL-based TWAS identified 99 cis-regulated expression genes associated with vertigo, such as *RAB3B* (*P*_TWAS_ = 0.017), *TMEM132E* (*P*_TWAS_ = 0.002), and *TCEA3* (*P*_TWAS_ = 0.037) (Supplementary Table 1). The sQTL-based TWAS identified 148 genes associated with vertigo, such as *MTERFD2* (*P*_TWAS_ = 0.003), *C12orf73* (*P*_TWAS_ = 0.027), and *SNRPC* (*P*_TWAS_ = 0.003) (Supplementary Table 2). Notably, eight candidate common genes were shared in eQTL-based and sQTL-based TWAS, such as *PIGG* (*P*_eQTL_ = 0.011, *P*_sQTL_ = 0.009), *ZSWIM7* (*P*_eQTL_ = 0.023, *P*_sQTL_ = 0.017), and *PMS2P5* (*P*_eQTL_ = 0.033, *P*_sQTL_ = 0.031) (Supplementary Tables 1 and 2).

Fetal brain-based TWAS identified 20 cis-regulated expression genes associated with vertigo, such as *MXRA7* (*P*_TWAS_ = 0.006), *MSH3* (*P*_TWAS_ = 0.018), and *DHFR* (*P*_TWAS_ = 0.038) (Supplementary Table 3). Interestingly, no common gene in fetal brain-based TWAS was shared with eQTL and sQTL-based TWAS, which might highlight the differences for vertigo mechanism in RNA level between adult and fetus.

### The comparison of PWAS and TWAS highlighted two risk genes for vertigo

PWAS and TWAS highlighted several promising candidate genes for vertigo, and a series of comparisons thus to be performed between PWAS and TWAS results to explore the overlapped risk genes at the protein and RNA level, including eQTL-based TWAS, sQTL-based TWAS, and fetal brain-based TWAS. Proteome-wide candidate risk gene *RAB3B* was significantly overlapped in eQTL-based TWAS (*P*_TWAS_ = 0.017). *MTERFD2* was statistically significant between PWAS and sQTL-based TWAS result (*P*_TWAS_ = 0.003). However, no proteome-wide significant genes were supported by fetal brain-based TWAS results.

### Colocalization analysis of common vertigo risk genes between PWAS and TWAS

Colocalization analysis based on the PP4 hypothesis which represents the posterior probability for coloc colocalization analysis hypothesis 4, that is, eQTL and GWAS signals are driven by the same common causal variant. The results indicated that the GWAS and pQTL signals were driven by the same risk variants, including *RAB3B* (PP4_banner_ = 0.007, PP4_ROSMAP_ < 0.001, PP4_RNAseq_ = 0.007), and *MTERFD2* (PP4_banner_ = 0.049, PP4_ROSMAP_ = 0.058, PP4_splicing_ = 0.006). In particular, *RAB3B* was detected to be associated with vertigo in two independent PWASs and eQTL-based TWAS, which strongly suggested that *RAB3B* might be a vital risk gene for vertigo.

### Chemical-related GSEA implicates potential pathogenesis for vertigo

As most chemicals have pathogenic effects in disease, we explored whether the chemicals in CTD can serve as potential pathogenic factors for vertigo. For the Banner PWAS gene list, 15 candidate chemicals were observed, such as glycidamide (*P* = 0.005), enzalutamide (*P* = 0.004), and dihydrotestosterone (*P* = 0.007). For the ROSMAP PWAS gene list, seven candidate chemicals were observed, such as nickel (*P* = 0.007), proanthocyanidins (PAC; *P* = 0.015), and irinotecan (*P* = 0.025) (Table 2). For the TWAS gene list, 22, 32, and 31 candidate chemicals were significantly enriched in fetal brain, RNA-seq and splicing data, respectively. Notably, three common chemicals overlapped in RNA-seq and splicing TWAS results, including fipronil, phenylpropanolamine, and fenbuconazole (Supplementary Table 4).

**Table 2:** Chemical-related gene set enrichment analysis for vertigo based on PWAS results

Chemical name	Banner		ROSMAP	
	NES	*P*	NES	*P*
Glycidamide	2.482	0.005	−1.926	0.974
Aldehyde	1.619	0.049	0.552	0.296
Menthol	1.725	0.041	0.423	0.336
Nimesulide	1.709	0.040	−0.183	0.574
Sirolimus	1.779	0.038	1.228	0.115
Enzalutamide	2.397	0.004	−0.160	0.573
Tunicamycin	1.744	0.041	−0.395	0.649
Vitallium	1.910	0.027	1.354	0.089
Dimethyloctadecan	1.739	0.041	−0.180	0.570
Belinostat	1.908	0.026	−0.096	0.535
Carcinogens	2.108	0.012	0.303	0.388
Carmustine	1.762	0.039	0.563	0.287
Clothianidin	2.130	0.015	0.392	0.351
DEET	2.100	0.011	0.766	0.228
Dihydrotestosterone	2.362	0.008	0.648	0.260
Gasoline	−0.020	0.507	1.743	0.040
Proanthocyanidins	−0.191	0.574	2.160	0.015
GSK-J4	1.012	0.156	2.180	0.015
Irinotecan	−0.652	0.738	1.954	0.025
Nickel	0.001	0.497	2.421	0.007
Oxazolone	0.086	0.475	1.806	0.032
Benzo(a)pyrene	−1.623	0.949	1.672	0.047

Dimethyloctadecan = 2-amino-14,16-dimethyloctadecan-3-ol; PWAS = proteome-wide association study; ROSMAP = Religious Orders Study and Rush Memory and Aging Project; NES = Normalized Enrichment Score.

### GO and pathway enrichment analysis

GO annotation and pathway analysis were accomplished to further reveal the pathogenesis of vertigo. A total of 15 significant terms were identified by using significant genes from PWAS and TWAS. For example, CNS (*P_FDR_* = 2.84 × 10^−7^) and brain stem (*P_FDR_* = 0.039) in CMC splicing dataset-based TWAS, mitochondrion (*P_FDR_* = 3.9 × 10^−4^) in Banner dataset-based PWAS, cognitive function measurement (*P_FDR_* = 0.023) and serine metabolism (*P_FDR_* = 0.012) in fetal brain dataset-based TWAS (Supplementary Table 5).

## Discussion

In this study, we performed PWASs for vertigo by integrating human brain pQTL data and genome-wide associations. We identified three genes that regulate protein levels in the human brain that were associated with risk of vertigo by combining the results from the discovery and replication PWASs. Among the three candidate genes, *RAB3B* and *MTERFD2* were also validated in TWAS, strongly emphasizing the consistent findings at mRNA and protein levels. Chemical-related GSEA and GO/pathway enrichment analysis highlights that the pathogenesis of vertigo may be related to neuronal exocytosis and CNS development.

Our study suggesting that *RAB3B* and *MTERFD2* are highly risk genes whose expression and protein abundance are significantly associated with vertigo. *RAB3B* encodes a low molecular weight GTP-binding protein, which is involved in the exocytosis of synaptic vesicles and secretory granules in the CNS and the anterior pituitary cells.^41^ In addition, RAB3B may be involved in polarized transport of basolateral and tight junctional membrane proteins to the plasma membrane and the regulation of synaptic plasticity.^42,43^*RAB3B* has been confirmed to be involved in neurotransmitters and synapses, pituitary exocytosis, and immune function. In mouse models, Rab3B is required for long-term depression of inhibitory synapses in the hippocampus, short-term plasticity, and normal reverse learning.^44^ As a key intracellular signalling molecule, *RAB3A* is highly expressed in the brain and can control the downstream exocrine secretion of other calcium-dependent processes in anterior pituitary cells.^45^ RAB3B immunoreactivity plays an important role in human pituitary adenoma,^46^ possibly reflecting the number of secretory granules and exocytosis activity.^47^ In addition, GO and KEGG pathway analysis also found that vertigo was involved in the biological function of neurons. For example, regulation of short-term neuronal synaptic plasticity in Banner PWAS, cell proliferation in the midbrain, commissural neuron axon guidance in RNA-seq TWAS, neurotransmitter secretion, and GABAergic synapse in splicing TWAS.

*MTERFD2*, also known as *MTERF4*, is another vertigo risk gene identified in this study that belongs to the component of the mitochondrial transcription termination factor (MTERF) family. Colocalization of EGFP-MTERFD2 fusion protein suggested that MTERFD2 targeting mitochondria exhibit a dynamic expression pattern during embryogenesis and might play an important role in organ differentiation.^48^ MTERFD2 has been shown to recruit ribosomal RNA through NSUN4 to regulate ribosomal biogenesis.^49^ It was found that *MTERFD2* also plays a vital role in mitochondrial regulation and neurodegenerative disease. Ye *et al*. found that the overexpression of *MTERFD2* in SH-SY5Y cells partially alleviated 1-methyl-4-phenylpyridinium induced mitochondrial dysfunction, and proposed that *MTERFD2* might be the trigger factor of the pathogenesis of Parkinson’s disease induced by environmental toxins.^50^ Wang *et al*. emphasized that *MTERFD2* plays a key role in the pathogenesis of Alzheimer’s disease by inhibiting ADAM10 in HEK293-APPswe cells and promoting amyloidogenic processing of β-amyloid precursor protein.^51^ Besides, GO annotation also found that vertigo is involved in the biological function of mitochondrial regulation, such as mitochondrion in Banner PWAS. Among the three genes overlapped in Banner and ROSMAP PWAS, *RAB3B* and *MTERFD2* were also verified by TWAS to be associated with vertigo at mRNA-level, emphasizing that genetic risk variants likely confer risk of vertigo by regulating messenger RNA expression and protein abundance of these genes.

In particular, *MGST1* and *SPRYD4* were only recognized by the PWAS rather than TWAS, highlighting the additional insights provided by focusing brain proteins directly. MGST1 protein is ubiquitous in human tissues and cell lines and is located in the endoplasmic reticulum and outer mitochondrial membrane, playing a protective role against membrane oxidative stress.^52^ It was found that *Mgst1* deletion in mice resulted in embryonic lethal and impaired haematopoietic function, emphasizing that *Mgst1* is essential for embryonic development and haematopoietic function in vertebrates.^53^*SPRYD4* has not been widely explored, but it has been recently found to have anti-cancer effects, such as acute myeloid leukemia^54^ and hepatocellular carcinoma.^55^ In our GO annotation, glutathione peroxidase activity was also found to involve in vertigo by Banner PWAS. Notably, all three proteome-wide significant genes were not specified by the original vertigo GWAS, highlighting the ability of PWAS in exploring vertigo risk genes.

Chemical-related GSEA identified multiple neurotoxic chemicals that may be associated with vertigo risk, such as nickel, glycidamide, fipronil. Nickel is a potential risk chemical for vertigo identified by our ROSMAP PWAS and has also been shown to cause vertigo, with toxicity manifesting in affecting T cell systems and inhibiting the activity of natural killer cells.^56^ Thus, environmental and occupational exposure to nickel is a potential risk factor for human brain dysfunction and neurological symptoms.^57^ In addition, GSEA based on TWAS results of RNA-seq and splicing jointly identified a potential risk chemical, fipronil, which is a member of the phenylpyrazole class of insecticide and has broad spectrum activity.^58^ Fipronil interferes with the GABAergic system, affecting brain growth and neural development.^59^ Lassiter *et al.* found that fibronil caused oxidative stress in undifferentiated neuronal PC12 cells and subsequently inhibited DNA and protein synthesis.^60^ Ki *et al.* subsequently indicated that fipronil-induced neuronal apoptosis was mediated by reactive oxygen species production.^61^

Conversely, our chemical-related GSEA have also identified some chemicals that might play a protective role in vertigo, such as PAC and piroxicam. PAC was a potential therapeutic chemical for vertigo identified by our ROSMAP PWAS and was also widely distributed in common foods including cereals, fruits, vegetables, and wines, and belonged to a universal group of plant polyphenols with their powerful antioxidant capacity and possible protective effects on human health.^62^ Chen *et al*. suggested that ethanol causes cognitive impairment and that the protective effect of PAC on ethanol-induced cognitive impairment may be due to its antioxidant and anti-inflammatory activities.^63^ Gong *et al*. found that lotus seedpod proanthocyanidins (LSPC) could prevent hippocampal neuron damage and reduce the expression of P53 protein in the hippocampus, suggesting that LSPC might be used to treat Alzheimer’s disease.^64^ Piroxicam was detected to be significantly enriched in fetal brain-based TWAS for vertigo, which could be converted into novel CNS stimulants or inhibitors through desulphurization, methylation, dehydrogenation, carboxylation, and carbonylation.^65^ Animal toxicity studies showed that Piroxicam significantly reversed oxidative stress induced by the mycotoxin 3-nitropropionic acid (3-NP) in mice, suggesting that Piroxicam protected mice from 3-NP-induced oxidative stress and behavioural changes in the brain.^66^ Mazumder *et al*. recommended the use of Piroxicam as the gold standard for the prevention of cerebral ischaemia (CI) neurodegeneration, since Piroxicam inhibits N-methyl-D-aspartate (NMDA) receptor, which are also associated with CI-induced neurodegeneration.^67^

Our study has several strengths. First, this study examined the mRNA and protein levels in vertigo through PWAS and TWAS. Second, GSEA provided potential risk chemicals and possible treatments for vertigo. There are several limitations of our study. First, the pre-computed reference weights of the ROSMAP and Banner brain proteome included both older and neurological disorder individuals, which may result in slight bias in our results. Besides, although tissue differences were controlled in the TWAS analysis, the weak anatomical correlation between vertigo and DLPFC could still lead to potential bias. Third, our method is a theoretical analysis of the sequencing data of European ancestors. Further studies of other genetic backgrounds and annotation analyses are needed to confirm our findings and reveal the underlying biological mechanisms of identified genes and chemicals in the development of vertigo.

In conclusion, we identified three high-confidence genes (*RAB3B*, *MTERFD2*, and *MGST1*) showed proteome-wide significant associations in the human brain with vertigo in two independent brain proteomes, strongly highlighting the underlying pathogenesis of these proteins in vertigo. Our study provides new insights into the genetically regulated protein abundance in vertigo and also highlights promising chemicals for further pathogenesis investigations and therapeutics research.

## Supplementary Material

fcac313_Supplementary_Data

## Abbreviations

3-NP =: 3-nitropropionic acid
Banner dataset =: Banner Sun Health Research Institute dataset
BSLMM =: Bayesian sparse linear mixed model
CI =: cerebral ischaemia
CMC =: CommonMind Consortium
CNS =: central nervous system
CTD =: Comparative Toxicogenomics Database
DLPFC =: dorsolateral prefrontal cortex
eQTL =: expression quantitative trait loci
ESs =: enrichment scores
GO =: gene ontology
GSEA =: gene set enrichment analysis
GWAS =: genome-wide association study
LD =: linkage disequilibrium
LSPC =: lotus seedpod proanthocyanidins
NMDA =: N-methyl-D-aspartate
PAC =: proanthocyanidins
pQTL =: protein quantitative trait loci
PWAS =: proteome-wide association study
QC =: quality control
ROSMAP =: Religious Orders Study or the Rush Memory and Aging Project
Sd =: standard deviation
sQTL =: splicing quantitative trait loci
TWAS =: transcriptome-wide association study
WGS =: whole-genome sequencing
