# Supplementary material for: Integrated analysis of proteome-wide and transcriptome-wide association studies identified novel genes and chemicals for vertigo
Source: Brain Commun. 2022 Nov 28;4(6):fcac313. doi: 10.1093/braincomms/fcac313 (PMC9732855; doi:10.1093/braincomms/fcac313)
Supplement: fcac313_Supplementary_Data [file fcac313_supplementary_data.zip › Supplementary Material.docx]

**Supplementary material (Materials and Methods)**

For GWAS summary data of vertigo [^1^](#_ENREF_1), the Icelandic association analysis was adjusted for sex, county of origin, current age or age at death, blood sample availability for the individual, and an indicator function for the overlap of the lifetime of the individual with the time span of phenotype collection. Sex, age, and the first 20 principal components were adjusted for population stratification. The US association analysis was adjusted for sex, age, and the first 20 principal components. The Finngen association analysis was adjusted for sex, age, the genotyping batch, and the first ten principal components.

For two brain proteome reference datasets [^2^](#_ENREF_2), samples were randomized by age, sex, postmortem interval, cognitive diagnosis and pathologies into 50 batches before tandem mass tag labeling to minimize batch effects. To remove the effects of protein loading differences, each protein abundance was scaled with a sample-specific total protein abundance and log_2_-transformed the abundance. Next, poorly performing samples were identified and removed using iterative principal component analysis to remove samples with greater than four standard deviations from the mean of either the first or second principal component. Subsequently, regression was used to estimate and remove the effects of proteomic sequencing batch, mass spectrometry reporter quantification mode, sex, age at death, postmortem interval and the final clinical diagnosis of cognitive status from the proteomic profile.

For the three gene expression panels, RNAseq and Splicing expression weights were obtained from the DLPFC of 452 European individuals collected by the CommonMind Consortium, and detailed data information is available in the FUSION pipeline [^3^](#_ENREF_3). Fetal brain expression weights were obtained from brain tissue from 120 human fetuses [^4^](#_ENREF_4). SNP-weights for Ensembl gene and transcript-level expression were derived from genotyping and RNA sequencing data from human fetal brains aged 12-19 post-conception weeks [^5^](#_ENREF_5). Prior to deriving SNP-weights, gene- and transcript-level expression data were normalized and adjusted for sex, age, RIN, sequencing batch, 3 genotype principal components and 10 PEER factors. Expression weights for use in TWAS were derived using FUSION software. SNP-weights were only generated for genes and transcripts with significant evidence of cis-heritable expression at the default P-value (P < 0.01) in FUSION (1,351 Ensembl genes and 3,985 Ensembl transcripts) [^5^](#_ENREF_5).

**Reference**

1. Skuladottir AT, Bjornsdottir G, Nawaz MS. A genome-wide meta-analysis uncovers six sequence variants conferring risk of vertigo. *Communications biology*. Oct 7 2021;4(1):1148. doi:10.1038/s42003-021-02673-2

2. Wingo AP, Liu Y, Gerasimov ES, Gockley J, Logsdon BA. Integrating human brain proteomes with genome-wide association data implicates new proteins in Alzheimer's disease pathogenesis. *Nature genetics*. Feb 2021;53(2):143-146. doi:10.1038/s41588-020-00773-z

3. Fromer M, Roussos P, Sieberts SK, et al. Gene expression elucidates functional impact of polygenic risk for schizophrenia. *Nature neuroscience*. Nov 2016;19(11):1442-1453. doi:10.1038/nn.4399

4. Hall LS, Pain O, O'Brien HE, Anney R, Walters JTR, Owen MJ. Cis-effects on gene expression in the human prenatal brain associated with genetic risk for neuropsychiatric disorders. *Mol Psychiatry*. Jun 2021;26(6):2082-2088. doi:10.1038/s41380-020-0743-3

5. O'Brien HE, Hannon E, Hill MJ, et al. Expression quantitative trait loci in the developing human brain and their enrichment in neuropsychiatric disorders. *Genome biology*. Nov 12 2018;19(1):194. doi:10.1186/s13059-018-1567-1
