# Supplementary material for: Integrated analysis of proteome-wide and transcriptome-wide association studies identified novel genes and chemicals for vertigo
Source: Brain Commun. 2022 Nov 28;4(6):fcac313. doi: 10.1093/braincomms/fcac313 (PMC9732855; doi:10.1093/braincomms/fcac313)
Supplement: fcac313_Supplementary_Data [file fcac313_supplementary_data.zip › Supplementary_Table_legends.25.11.docx]

**Supplementary Tables**

Supplementary Table 1. Transcriptome-wide Significant Genes for Vertigo by eQTL-based TWAS

Supplementary Table 2. Transcriptome-wide Significant Genes for Vertigo by sQTL-based TWAS

Supplementary Table 3. Transcriptome-wide Significant Genes for Vertigo by Fetal brain-based TWAS

Supplementary Table 4. Chemical-related gene set enrichment analysis for vertigo based on TWAS results

Supplementary Table 5. GO and KEGG pathway enrichment analysis for vertigo based on PWAS and TWAS results
